# A more comprehensive investigation of disability and associated factors among older adults receiving home-based care in rural Dongguan, China

**DOI:** 10.1186/s12877-018-0852-x

**Published:** 2018-07-06

**Authors:** Yaping Liang, Xiaojia Xu, Mingjuan Yin, Yulian Li, Yan Zhang, Lingfeng Huang, Jindong Ni

**Affiliations:** 1 Department of Epidemiology and Biostatistics, Dongguan Key Laboratory of Environmental Medicine, School of Public Health, Guangdong Medical University, Dongguan, China; 2 Da Lang Community Health Service Center, Dongguan, China

**Keywords:** Comprehensive ability, Health-related behavior, Multimorbidity

## Abstract

**Background:**

No previous study has evaluated disability in older persons according to the International Classification of Functioning, Disability and Health Framework guidelines. We conducted a more comprehensive investigation of disability and associated factors among older adults receiving home-based care in rural Dongguan, a city in the central Guangdong Province of Southern China.

**Methods:**

A total of 819 individuals aged ≥60 years were recruited from Dongguan home-based care system of via a two-stage selection process. We interviewed participants and assessed their ability level using the Ability Assessment for Older Adults, which defined by a combination of activity of daily living, sensory perception, mental status and social involvement. Conditional probability and Logistic regression approaches were used to assess the strength of association between each pair of conditions. Factors significantly associated with disability were identified via *χ*^2^ tests and multinomial ordinal logistic regression.

**Results:**

Of the 819 included participants (mean age 87 ±4.7 years), 75.5% were female, 76.7% had any disability, and 62.3% had a mild disability. The occurrence of any deficits significantly increased the likelihood of the co-occurrence of other deficits (odds ratio [OR] > 1, *P* < 0.05), with the lowest prevalence odds ratio observed among individuals with sensory and communication deficiency (OR: 2.99; 95% confidence interval [CI]: 2.21–4.05). Multivariable ordinal logistic regression analysis indicated that physical activity (OR: 0.96; 95% CI: 0.93–0.99), sedentary behavior (OR: 1.25; 95% CI: 1.13–1.38), not watching television (OR: 1.7; 95% CI: 1.07–2.72) and age (OR: 1.09; 95% CI: 1.02–1.17) were significantly associated with disability.

**Conclusions:**

Impairment of ADL, sensory perception, mental status or social involvement increased the likelihood of risk of the co-occurrence of other deficits. Comprehensive disability among older adults receiving home-based care is associated with age, sedentariness, physical activity and TV viewing.

**Electronic supplementary material:**

The online version of this article (10.1186/s12877-018-0852-x) contains supplementary material, which is available to authorized users.

## Background

The average age of population is increasing [1], primarily due to a marked decrease in mortality rates, resulting in a significant increase in the proportion of individuals aged older than 65 years and, in particular, an increase in individuals who are greater than 85 years old. Concomitantly, family structures are changing, with a trend of having fewer children. Additionally, a growing incidence of chronic disease and functional impairment places increased demands on home-based care services for the older adults [2, 3]. The provision of home-based care for older citizens is a public service in China that is delivered by social workers and includes provision of care to older adults who live alone, who are unable to care for themselves or who are aged 80 years or older.

Population-based study indicated that 18.3% of older persons suffering from disability [4]. Disability is linked to adverse health outcomes such as higher healthcare costs, admission to hospital care, reduced quality of life (QOL) and mortality. The effects of disability have been assessed by a multitude of studies, with the Instrument Activities of Daily Living (IADL) and Activities of Daily Living (ADL) to evaluate disability [5]. However, IADL and ADL reflect limitations on daily living activity. As stated by the World Health Organization (WHO), disability is an umbrella term that considers impairment and limitations in activity and in participation [6], all of which are associated with a reduction in the QOL of older adults [7, 8]. A recent systematic review concluded that no study has evaluated the disability through a questionnaire covered all essential dimensions proposed by International Classification of Functioning, Disability, and Health framework [9]. Therefore, what we seriously lack is more comprehensive data about disability covering impairments, activity limitations, and participation restrictions.

Multi-morbidity was defined as the co-occurrence of two or more chronic conditions in an individual, it often occurs in older adults, with a prevalence ranging from 6.4 to 76.5% among individuals aged 60 years or more [10]. Most previous studies defined an association between disability and a single chronic disease or various multi-morbidity combinations of chronic disease [11–13]. A positive correlation between multi-morbidity and disability, as defined by ADL/IADL, has been reported [14], though this study was based on an older adults population aged 80 or older with 2085 individuals. Moreover, having multiple chronic conditions has a significant negative effect on cognitive impairment [15, 16]. However, it is unknown if multimorbidity impacts adversely upon the more comprehensive disability.

Increasing longevity and changes in traditional family structures imply that the proportion of older individuals receiving home-based care will increase. This population is more likely to be disability and to be affected by chronic disease, health-related behavior and other factors. The aim of this study was to assess the association of inclusive disability characteristics and the potential association between multimorbidity, health-related behavior and disability in older community residents receiving home-based care.

## Methods

### Study design

**Fig. 1:**
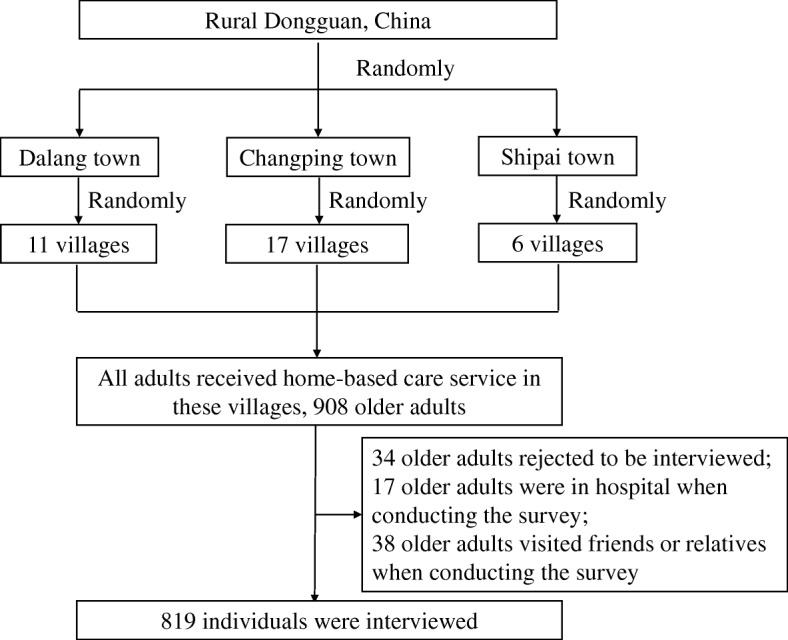
The flow diagram of participants selection

A cross-sectional household survey, based on a representative sample of older adults who aged 60 years or older was performed. Participants were recruited from the home-based care service in Dongguan, a city in the central Guangdong Province of South China. A two-stage recruitment process was employed. Initially, three towns were selected at random from within Dongguan City. Thereafter, 11, 17 and six villages were randomly and proportionally selected from the three towns. Older people living in any of these villages were included in the study. Eighty-nine participants were either absent when conducting the survey or unwilling to be interviewed. In total, 819 eligible people from 34 villages and under the care of social workers, were evaluated (details see Fig. 1).

Face-to-face interviews were conducted at participants’ homes between March and October of 2016. Family members or caregivers completed the questionnaire if the interviewee could not respond due to visual, hearing or cognitive impairment. Interviewers were medical students or graduate students who were trained on interviewing skills and the purpose of this study prior to performing interviews. Informed consent was obtained from each participant or, where appropriate, from their primary caregiver.

### Definition of ability level

The primary classification of individuals used in this study is ability level, which was calculated by a computer-based algorithm according to a combination of activity of daily living (ADL) status, mental status, sensory and communication ability and social involvement. This parameter was determined in line with the guidelines to assess the ability level for older adults, released by the Ministry of Civil Affairs of the People’s Republic of China (see in Additional file 1) [17]. These criteria have a Cronbach’s *α* of 0.725 for reliability of a psychometric test, indicating an acceptable consistency when applied to the Jiangsu older adults population [17]. Four categories of individual were classified, namely able, mild disability, moderate disability and severe disability.

The ADL status was measured by the Barthel index [18]. Participants were asked if they had difficulty in performing the following ten activities: eating, bathing, dressing, grooming, controlling bowel function, controlling bladder function, using a toilet unaided, transferring, walking and stair climbing. Scores range from 0 to 100, with higher scores indicating better performance in the activities of daily living.

We evaluated the mental status of participants by Mini-Cog assessment [19], in combination with aggressive behavior and depressive symptoms. The Mini-Cog test consisted of a clock drawing test and recall of three unrelated items. The score of the aggregate of these evaluations ranges from zero to six. Score zero indicates a normal cognitive state and score six designated a severe disability status.

The assessment of sensory and communicative ability was defined by the interRAI Acute Cognitive Performance Scale (CPS) [20], which is based on the awareness, communication ability, vision and hearing abilities of Geriatrics. A scaled, parameter based algorithm was used to determine CPS.

The social involvement of each participant was evaluated using the intellectual disability rating scale for adults [21]. Scores range from 0 to 20, with lower scores indicating good social involvement. The questionnaire evaluated five-items: independence in living, performing work, social interactions, identifying interpersonal relationships and orienting time and space. A limit of 8 and above indicating ‘moderately impairment’ was employed.

### Multi-morbidity

We asked all participants if they suffered from the following 14 common conditions that have a significant impact in the functional status of the older adults: hypertension, coronary heart disease, cardiac arrhythmia, diabetes, chronic gastritis, cataracts, arthritis, hyperlipidemia, stroke, cancer, chronic kidney disease, Parkinson’s disease, bronchitis and anemia. Participants self-reporting two or more chronic conditions were deemed to have multi-morbidity.

### Social and environmental variables

The physical activity in hours per week, smoking status and alcohol consumption were recorded, as was the number of hours viewing TV per day. Social demographics recorded including age, gender, income, marital status, education level and living arrangement. Living arrangement was categorized as living alone, living with family or living with other relatives or with non-relatives. Educational level was classified as illiterate, completion of primary education or completion of secondary education and above. Annual income was classified as 0 to 5000 ¥, 5000 to 10,000 ¥, and greater than 10,000 ¥. Data were collected by questions listed in Additional file 1.

### Statistical analysis

Analyses were performed using the SPSS 15.0 statistical software package (SPSS, Chicago, IL, USA). The frequency, mean and standard deviation of all variables were determined. The conditional probability of each disability in relation to every other disability was calculated to evaluate the prevalence of one impairment in relation to other impairments. Logistic regression was performed to assess the strength of association between each pair of conditions. The chi squared test and Kruskal-Wallis *H* test was used to determine univariate association of each socio-demographic factor. Multinomial ordinal logistic regression was employed to identify factors associated with disability. Odds ratios (OR) and 95% confidence intervals (95% CI) were obtained for every case. Statistical significance was set at *p* < 0.05.

## Results

### Sample characteristics

The survey was completed by 819 respondents aged 60 years and older, out of 908 sample requests (response rate of 90.1%). Respondents had a mean age of 87 (4.7) years, with 76.5% female. Older adults living alone accounting for 62.3%. The percentage of the study population achieving primary education or higher education was 30.1%. Overall, 34.3% participants reported multi-morbidity and 79.1% reported at least one chronic condition. A summary of the demographic characteristics and multi-morbidity prevalence is presented in Table 1.

**Table 1:** Characteristics of individuals in home-based care system in Dongguan, China

Characteristic	Total
Study population, *n (%)*	819
Age (years), *mean (SD)*	87 (4.7)
Female, *n (%)*	627 (76.5)
Living arrangement, *n (%)*	
Living alone	510 (62.3)
With family	276 (33.7)
With other relatives/non-relatives	33 (4.0)
Education, *n (%)*	
Illiterate	561 (68.5)
Primary	247 (30.1)
Secondary and above	11 (1.4)
Marital status, *n (%)*	
Married	361 (44.1)
Single/ Divorced/ Widowed	458 (55.9)
Income (Ұ), *n (%)*	
0~	310 (37.8)
5000~	353 (43.2)
10,000~	156 (19.0)
If smoking, *n (%)*	
Yes	84 (10.3)
No	735 (89.7)
If drinking, *n (%)*	
Yes	33 (4.0)
No	786 (96.0)
Number of diseases, *n (%)*	
0	171 (20.9)
1	367 (44.8)
2	198 (24.2)
3+	83 (10.1)

### Association between inclusive disability characteristics

Three primary characteristics; impairment in ADL, reduced cognitive ability and debility in sensory and communication perception, significantly increased co-occurrence with every other parameter analyzed in this study, with the greatest effect seen between impairment of social involvement and primary characteristics of ageing (ADL (OR: 7.06; 95% CI: 5.16, 6.98); sensory acuity (OR: 3.09; 95% CI: 2.29, 4.17) and communication perception (OR: 5.83; 95% CI: 4.27, 7.94). Reduced social involvement enhanced the risk of impairment of all other conditions analyzed, with the greatest risk of disability in ADL (OR: 7.06; 95% CI: 5.16, 6.98). All prevalence odds ratios for the co-occurrence of impairment in ADL, reduced cognitive ability, reduced social involvement and debility in sensory and communication perception are presented in Table 2.

**Table 2:** The conditional probabilities (%) and prevalence odds ratio (95% CI) for analyzed dimensions (The percentages represent the frequency of impaired cases in dimensions described in the columns among those with the conditions given in the rows)

		ADL	Mental status	Sensory and communication	Social involvement
ADL	Y (%)		64.1%	58.9%	72.2%
	N (%)		37.0%	22.3%	26.9%
	OR		3.03 (2.24 to 4.10)	4.96 (3.62 to 6.78)	7.06 (5.16 to 9.68)
Mental status	Y (%)	68.5%		52.3%	65.4%
	N (%)	46.9%		28.3%	37.9%
	OR	3.03 (2.24 to 4.10)		2.99 (2.21 to 4.05)	3.09 (2.29to 4.17)
Sensory and communication	Y (%)	76.6%	67.8%		75.3%
	N (%)	40.0%	41.3%		34.3%
	OR	4.96 (3.62 to 6.78)	2.99 (2.21 to 4.05)		5.83 (4.27 to 7.94)
Social involvement	Y (%)	77.4%	65.4%	62.2%	
	N (%)	32.4%	37.9%	22.0%	
	OR	7.06 (5.16 to 9.68)	3.09 (2.29 to 4.17)	5.83 (4.27 to 7.94)	

### Factors associated with disability

Table 3 presents the distribution of the level of disability, defined as able or as mild, moderate or severe disability, in relation to demographic characteristics, health-related behavioral factors and multi-morbidity. Compared with participants who were married, individuals who were single, divorced or widowed were more likely to be disability (*p* = 0.023). The prevalence of disability was significantly more common in people who lived alone or with non-relatives than in people living within their family (86.7% in individuals living with non-relatives, 77% when living alone, 74.5% when living with family (*p* = 0.001). Disability was more frequently reported among people suffering from multi-morbidity than those with no chronic disease (*p* = 0.022). The prevalence of disability was also related to age (*p* < 0.001), sedentariness (*p* < 0.001), number of medicaments required (*p* = 0.029), hours watching TV (*p* = 0.001) and extent of physical activity (*p* < 0.001). Education level, income, smoking status and alcohol consumption were not significantly associated with disability.

**Table 3:** Univariate associations between disability and multimorbidity and health-related behavior

	Comprehensive ability level					
	Undamaged, *n* = 191, (%/M)	Mild damaged, *n* = 510, (%/M)	Moderate damaged, *n* = 55, (%/M)	Severe damaged, *n* = 63, (%/M)	*χ* ^*2*^	*p*
Educational level						
Illiterate	25.9	62.9	5.2	6.0		
Primary	48.8	46.1	3.0	2.1		
Secondary and above	63.8	34.5	1.7	0		
					10.55	0.103
Marital status						
Married	28.0	58.5	9.8	3.7		
Single/divorced/widowed	22.4	63.6	5.6	8.5		
					9.52	0.023
Gender						
Female	21.3	64.0	6.5	2.8		
Male	30.1	56.6	7.5	5.8		
					6.73	0.081
Living arrangement						
Living alone	23.0	63.4	6.2	7.5		
With spouse/children	25.5	62.5	6.9	5.0		
With others	13.3	46.7	13.3	26.7		
					22.24	0.001
Income						
0~	32.8	58.5	5.0	0.037		
5000~	29.3	60.0	5.8	4.9		
10,000~	54.9	39.6	1.2	4.3		
15,000~	50.3	47.9	0.6	1.2		
					67.9	0.136
Multimorbidity						
0	31.8	51.4	10.8	6.1		
1	23.1	64.2	5.9)	6.8		
2	20.6	68.6	5.7)	5.1		
3+	15.3	69.4	4.2)	11.1		
					19.46	0.022
Smoking						
Current smoker	31.1	64.4	4.4	0.0		
Ex-smoker	25.7	62.2	6.6	5.6		
Non-smoker	19.4	70.4	6.5	3.7		
					6.05	0.418
Drinking						
Yes	52.1	51.3	3.2	2.3		
No	43.1	45.8	2.1	0		
					2.44	0.48
Watching TV						
Yes	30.8	60.2	5.6	3.4		
No	14.2	72.2	7.7	5.9		
					16.23	0.001
Age	85	86	89	86	40.31	< 0.001
Number of drug treatments	1	1	1.5	1	9.06	0.029
Physical activity, hours/week	12.25	7	0	0	16.73	< 0.001

After fitting the multinomial ordinal logistic regression, age and sedentariness remained significantly and positively associated with disability (OR: 1.09, 95% CI: 1.02 to 1.17 and OR: 1.25, 95% CI: 1.13 to 1.38 respectively). Living arrangement, marital status and multi-morbidity were not associated with disability. Finally, a lack of physical activity and not watching TV were significantly associated with disability (OR: 0.96, 95% CI: 0.93 to 0.99 and OR: OR: 1.7, 95% CI: 1.07 to 2.72 respectively). The results were shown in Table 4.

**Table 4:** Multiple ordinal logistic regression analyses of significant factors associated with disability among participants

	Comprehensive ability level		
	OR	95% *CI*	*p*
Age	1.09	1.02, 1.17	0.013
Sedentariness, hours/week	1.25	1.13, 1.38	< 0.001
Physical activity, hours/week	0.96	0.93, 0.99	0.003
Watching TV			
Yes	1		
No	1.7	1.07, 2.72	0.024

## Discussion

Overall, 76.7% of 819 individuals aged 60 years or over reported that they had a disability. Impairment in ADL, mental status, sensory perception, ability to communicate, and social involvement significantly increased the risk of the co-occurrence of other impairment. Age and sedentary behavior was negatively associated with severe disability, with sedentary behavior and not watching TV viewing increasing the likelihood of disability.

Our survey revealed that more than three quarters of older adults receiving home-based care have an impairment in comprehensive ability, with 7% of participants having impairment to the extent that they were fully dependent upon others for basic needs. Less than one quarter of respondents remain in good health and are fully independent. The result is in agreement with results of a study focusing on centenarians in Hainan [22]. Additionally, research conducted in Chongqing reported that 65.6% of centenarians had disability associated with ADL [23]. The data from the China Health and Retirement Longitudinal Study (CHARLS) in 2013 [24] found that the overall prevalence of disability among adults aged 80 years or more was 21.9%. The prevalence of disability in our study is higher than other recent studies [25, 26], which may result from the following considerations. Firstly, previous studies assessed disability according to ADL and IADL [9, 27], without considering the impact of impairment of sensory perception, social participation and mental status on compromising independent living status. This may result in under-estimation of the prevalence of debility. Secondly, the sample population in this study consisted of elder adults who either lived alone, were dependent on external support or who were aged 80 years or older, which is more likely to be disability.

Our study found that 34.3% of participants experienced multi-morbidity. This is lower than the findings of a meta-analysis of studies on the prevalence of multi-morbidity in developing countries, which ranged from 55 to 89% [28]. The prevalence of older persons reporting multi-morbidity in our analysis was higher as compared with several relevant studies of Chinese older adults population [14, 29–31]. The differences in the prevalence of multi-morbidity between studies might arise from significant differences in the criteria used to define multi-morbidity. In particular, self-reporting of chronic disease was employed in our study; consequently, this may more accurately reflect the incidence of multi-morbidity in an aged population.

Previous studies have reported a highly significant correlation between age and disability in older adults [32–34]. In our study, we found that increasing age was associated with more extensive disability. This is in accordance with age-related loss of physical prowess with age [35]; where the incidence of almost all chronic conditions increases with age [36, 37]. In particular, the occurrence of chronic conditions such as presbyopia, loss of strength, balance and osteoporosis may have a considerable impact on functional independence [38].

The results suggested that the participants who suffered from any of the tested geriatric troubles had obviously higher risk of co-occurrence of other listed deficits, As far as we know, no relevant research estimating the prevalence odds ratio for multiple geriatric deficits in a Chinese older adults sample has been conducted. The result was in line with that of a cross sectional study in Poland.

Theses information about the prevalence and risk of co-occurrence of the listed conditions seems crucial for both social workers and health-care policy makers. Health care providers should begin with extensive screening and preventive strategies to address the complex geriatric conditions.

Our study confirmed that older people receiving home-based care in rural Dongguan who were less physically active had a higher likelihood of disability. This is in agreement with the recognition that physical activity is beneficial to the physical health and independence of ageing individuals. A significant negative correlation between ADL score and physical activity was also reported in Sardinian older adults [39]. Additionally, previous studies in the United States and in Korea [40, 41] also indicated that lack of physical activity is associated with a reduction in the daily quality of life. So, older adults should be suggested to do some exercise or manageable housework to maintain the function of muscle and undamaged ability.

Our study indicated that less sedentary behavior significantly reduces the risk of disability. This is in agreement with the findings of Eileen Rillamas-Sun et al., [42], which found that older women who were immobile for more than 10 h a day were ten-fold more likely to be disability than women immobile for less than 5 h per day. In addition, sedentary behavior is linked to multi-morbidity [43] and with cognitive decline [44]. It is vital to call for the older persons to reduce sedentary time for lowering the prevalence of disability.

Older individuals who did not watch TV reported a higher incidence of disability in our study. This is in contrast to the findings of Wu et al., [23] who found no significant association between disability and watching TV in Centenarians. In contrast, a strong association between TV viewing time and disability was observed in an American study [45]. These contrasting findings may be a consequence of participants in different studies being of a different mean age, as the sample population of the American National Institutes of Health (NIH)-American Association of Retired Persons (NIH-AARP) Diet and Health study had a mean age of 87 years, while the population considered in Wu et al. involved individuals aged over 100 years old. Moreover, we employed further considerations of disability, in addition to ADL as used by other studies, as we also considered mental status, sensory perception and social involvement in relationship to disability. Pragmatically, people with disability are likely to have reduced or no ability to watch TV, due to either an inability to see or to hear, or both. Moreover, the American study was a longitudinal study, with TV viewing time collected at baseline and disability conditions recorded at follow-up. In contrast, we employed a cross-sectional investigation where data on time spent watching TV and level of disability was collected at the same time. In our study, it may be that older people who spent more time watching TV are at high-risk of developing disability in the future, even although they were in good health when sampled. Moreover, the oldest people in our sample are more likely to be disability and also to have no access to TV.

The prevalence of disability in our study among older people receiving home-based care system was higher than in other national studies. However, 82% of all people with disability in our study self-reported mild disability that was unlikely to make a significant impact on their quality of life. It is therefore appropriate to identify and respond to the needs of older adults with different levels of disability with the aim to provide them with appropriate care and to prevent further deterioration in their health. Social workers who take care of older people should be trained on how to care for the varying degrees of disability of older adults and to provide appropriate intervention measures to reduce the development of further disabilities. Overall, this would provide older people with an improved, more beneficial and targeted service.

There are limitations to our study. As we performed a cross-sectional study, it is not possible to identify causal association between multi-morbidity, health-related behavior and disability. Further longitudinal studies on our sample population are therefore necessary. Secondly, only limited health characteristics were recorded, and in particular, the diet of our population, which is likely to impact on disability, was not included in our present survey. Thirdly, we conducted our study only within Dongguan city and consequently, concluding nationwide generalizations from this data might be limited when considering the diversity of customs, different climate zones and the imbalance of economic development throughout China.

## Conclusion

The prevalence of disability among older people receiving home-based care in rural Dongguan was relatively high. Older people with younger age and beneficial health-related behavior such as physical activity, less sedentary behavior and watching TV, are more likely to have a healthier comprehensive ability status. Besides, impairment of ADL, sensory perception, mental status or social involvement increased the likelihood of co-occurrence of other deficits. Early and targeted intervention measures should be organized to delay the course of disability.

## Additional file

Additional file 1: English language copy of questionnaire and interview guides. (DOC 106 kb)

## Abbreviations

ADL: Activities of daily living
CI: Confidence interval
CPS: Cognitive Performance Scale
IADL: Instrument Activities of Daily Living
OR: Odds ratio
QOL: Quality of life

## References

[1] WHO (2015). World report on ageing and health.

[2] Rodriguez-Manas L, Rodriguez-Artalejo F, Sinclair AJ (2017). The third transition: the clinical evolution oriented to the contemporary older patient. J Am Med Dir Assoc.

[3] Chen D. Study on community home-based care model in China. Shandong: Shan dong University; 2015.

[4] The fourth sampling survey of the living conditions of urban and rural residents in China issused by three departments [http://www.xinhuanet.com/health/2016-10/10/c_1119683376.htm].

[5] Barberger-Gateau P, Rainville C, Letenneur L, Dartigues JF (2000). A hierarchical model of domains of disablement in the elderly: a longitudinal approach. Disabil Rehabil.

[6] Disability. [http://www.who.int/topics/disabilities/en/].

[7] Ponce MS, Rosas RP, Lorca MB (2014). Social capital, social participation and life satisfaction among Chilean older adults. Rev Saude Publica.

[8] Elliott AF, McGwin G, Kline LB, Owsley C (2015). Vision impairment among older adults residing in subsidized housing communities. The Gerontologist.

[9] Yang M, Ding X, Dong B (2014). The measurement of disability in the elderly: a systematic review of self-reported questionnaires. J Am Med Dir Assoc.

[10] Hu X, Huang J, Lv Y, Li G, Peng X (2015). Status of prevalence study on multimorbidity of chronic disease in China: systematic review. Geriatr Gerontol Int.

[11] Forjaz MJ, Rodriguez-Blazquez C, Ayala A, Rodriguez-Rodriguez V, de Pedro-Cuesta J, Garcia-Gutierrez S, Prados-Torres A (2015). Chronic conditions, disability, and quality of life in older adults with multimorbidity in Spain. Eur J Intern Med.

[12] Ralph NL, Mielenz TJ, Parton H, Flatley AM, Thorpe LE (2013). Multiple chronic conditions and limitations in activities of daily living in a community-based sample of older adults in new York City, 2009. Prev Chronic Dis.

[13] Tinetti ME, McAvay GJ, Chang SS, Newman AB, Fitzpatrick AL, Fried TR, Peduzzi PN (2011). Contribution of multiple chronic conditions to universal health outcomes. J Am Geriatr Soc.

[14] Su P, Ding H, Zhang W, Duan G, Yang Y, Chen R, Duan Z, Du L, Xie C, Jin C (2016). The association of multimorbidity and disability in a community-based sample of elderly aged 80 or older in shanghai, China. BMC Geriatrics.

[15] Vassilaki M, Aakre JA, Cha RH, Kremers WK, St Sauver JL, Mielke MM, Geda YE, Machulda MM, Knopman DS, Petersen RC (2015). Multimorbidity and risk of mild cognitive impairment. J Am Geriatr Soc.

[16] Yates JA, Clare L, Woods RT, Cognitive F, Ageing Study W (2017). What is the relationship between health, mood, and mild cognitive impairment?. J Alzheimers Dis.

[17] Zhao YY, Ding YP, Li XW, Cui Y (2015). Classification and determination of the competence level of the elderly. Chin J Health Stat.

[18] Mahony FI, Barthel DW (1965). Functional evaluation: the BARTHEL index. Md State Med J.

[19] Scanlan J, Borson S (2001). The mini-cog: receiver operating characteristics with expert and naïve raters. Int J Geriatr Psychiatry.

[20] Wellens NI, Flamaing J, Tournoy J, Hanon T, Moons P, Verbeke G, Boonen S, Milisen K (2013). Convergent validity of the cognitive performance scale of the interRAI acute care and the mini-mental state examination. Am J Geriatr Psychiatry.

[21] Yu YP, Zhao JC, Cui P (1996). Study on the consistency of adult intellectual disability scale and the Wechsler adult intelligence scale. Shanghai Psychiatry.

[22] Yao Y, Zhao YL, Yang SS, Liu M, Wang JH, Wu L, Wang YY, Zeng J, Li J, Luan FX (2017). Status of daily life activities and respective risk factors among centenarian population in Hainan province. Chin J Epidemiol.

[23] Wu T, Lu L, Luo L, Guo Y, Ying L, Tao Q, Zeng H, Han L, Shi Z, Zhao Y. Factors associated with activities of daily life disability among centenarians in rural Chongqing, China: a cross-sectional study. Int J Environ Res Public Health. 2017;14(11):1364. 10.3390/ijerph14111364 PMC5708003 29120382

[24] Zhou XF, Ma YN (2017). Prevalence and impact factors of disability among elderly people in rural China. Chin J Public Health.

[25] Villarreal AE, Grajales S, Lopez L, Britton GB, Panama Aging Research I (2015). Cognitive impairment, depression, and Cooccurrence of both among the elderly in Panama: differential associations with multimorbidity and functional limitations. Biomed Res Int.

[26] Chen W, Fang Y, Mao F, Hao S, Chen J, Yuan M, Han Y, Hong YA (2015). Assessment of disability among the elderly in Xiamen of China: a representative sample survey of 14,292 older adults. PLoS One.

[27] Palmer M, Harley D (2012). Models and measurement in disability: an international review. Health Policy Plan.

[28] Marengoni A, Angleman S, Melis R, Mangialasche F, Karp A, Garmen A, Meinow B, Fratiglioni L (2011). Aging with multimorbidity: a systematic review of the literature. Ageing Res Rev.

[29] Wang XX, Lin WQ, Chen XJ, Lin YY, Huang LL, Zhang SC, Wang PX. Multimorbidity associated with functional independence among community-dwelling older people: a cross-sectional study in southern China. Health Qual Life Outcomes. 2017;15(1):73. 10.1186/s12955-017-0635-7 PMC5392938 28412945

[30] Wang R, Yan Z, Liang Y, Tan ECK, Cai C, Jiang H, Song A, Qiu C. Prevalence and patterns of chronic disease pairs and multimorbidity among older Chinese adults living in a rural area. PLoS One. 2015;10(9):e0138521 10.1371/journal.pone.0138521 PMC4578976 26394368

[31] Kunna R, San Sebastian M, Stewart Williams J. Measurement and decomposition of socioeconomic inequality in single and multimorbidity in older adults in China and Ghana: results from the WHO study on global AGEing and adult health (SAGE). Int J Equity Health. 2017;16(1):79. 10.1186/s12939-017-0578-y PMC5433064 28506233

[32] Huang X, Yang H, Wang HH, Qiu Y, Lai X, Zhou Z, Li F, Zhang L, Wang J, Lei J (2015). The association between physical activity, mental status, and social and family support with five major non-communicable chronic diseases among elderly people: a cross-sectional study of a rural population in southern China. Int J Environ Res Public Health.

[33] Hacihasanoglu R, Yildirim A, Karakurt P (2012). Loneliness in elderly individuals, level of dependence in activities of daily living (ADL) and influential factors. Arch Gerontol Geriatr.

[34] Qian JH, Wu K, Luo HQ, Cao PY, Ren XH (2016). Prevalence of loss of activities of daily living and influencing factors in elderly population in China. Zhonghua liu xing bing xue za zhi = Zhonghua liuxingbingxue zazhi.

[35] Piotrowicz K, Pac A, Skalska AB, Chudek J, Klich-Raczka A, Szybalska A, Michel JP, Grodzicki T (2016). Clustering of geriatric deficits emerges to be an essential feature of ageing - results of a cross-sectional study in Poland. Aging.

[36] Ha NT, Le NH, Khanal V, Moorin R (2015). Multimorbidity and its social determinants among older people in southern provinces, Vietnam. Int J Equity Health.

[37] Chung RY, Mercer S, Lai FT, Yip BH, Wong MC, Wong SY (2015). Socioeconomic determinants of multimorbidity: a population-based household survey of Hong Kong Chinese. PLoS One.

[38] WHO (2011). World report on disability.

[39] Pes GM, Dore MP, Errigo A, Poulain M. Analysis of physical activity among free-living nonagenarians from a Sardinian Longevous population. J Aging Phys Act. 2018;26(2):254-8. 10.1123/japa.2017-0088 28714795

[40] Kim H, Lee T, Lee S, Kim K, Lee S, Kam S, Ahn S, Cho J, Ory MG (2012). Factors associated with ADL and IADL dependency among Korean centenarians: reaching the 100-year-old life transition. Int J Aging Hum Dev.

[41] Gerst K, Michaels-Obregon A, Wong R (2011). The impact of physical activity on disability incidence among older adults in Mexico and the United States. J Aging Res.

[42] Rillamas-Sun E, LaMonte MJ, Evenson KR, Thomson CA, Beresford SA, Coday MC, Manini TM, Li W, LaCroix AZ. The influence of physical activity and sedentary behavior on living to age 85 years without disease and disability in older women. J Gerontol A Biol Sci Med Sci. 2017; 10.1093/gerona/glx222. 10.1093/gerona/glx222 PMC6175029 29165626

[43] Vancampfort D, Stubbs B, Koyanagi A (2017). Physical chronic conditions, multimorbidity and sedentary behavior amongst middle-aged and older adults in six low- and middle-income countries. Int J Behav Nutr Phys Act.

[44] Wheeler MJ, Dempsey PC, Grace MS, Ellis KA, Gardiner PA, Green DJ, Dunstan DW (2017). Sedentary behavior as a risk factor for cognitive decline? A focus on the influence of glycemic control in brain health. Alzheimers Dement (N Y).

[45] DiPietro L, Jin Y, Talegawkar S, Matthews CE. The joint associations of sedentary time and physical activity with mobility disability in older people: the NIH-AARP diet and health study. J Gerontol A Biol Sci Med Sci. 2018;73(4):532-8. 10.1093/gerona/glx122 PMC5861886 28958064

