# Supplementary material for: A more comprehensive investigation of disability and associated factors among older adults receiving home-based care in rural Dongguan, China
Source: BMC Geriatr. 2018 Jul 6;18:158. doi: 10.1186/s12877-018-0852-x (PMC6034336; doi:10.1186/s12877-018-0852-x)
Supplement: Supplementary file 1 — English language copy of questionnaire and interview guides. (DOC 106 kb) [file 12877_2018_852_MOESM1_ESM.doc]

**The translation of questionnaire**

**NO.：**

A1. **Gender:** 1 male 2 female

A2. **Birthyear：**

A3. **Educational level:** 1 Illiterate 2 Primary 3 Secondary and above

A4. **Marital status:** 1 Married 2 Single/divorced/widowed

A5. **Living arrangement:** 1 Living alone 2 With spouse/children 3 With others

A6. **Income(Yuan)/per year:** 1 0~ 2 5000~ 3 10000~ 4 15000~

A7. **If you suffering from the following diseases:** 1 none 2 hypertension 3 coronary heart disease (CHD) 4 arrhythmia 5 diabetes mellitus 6 digestive system disease 7 Bone and joint disease 8 hyperlipaemia 9 cerebrovascular disease 10 cancer 11 chronic kidney disease 12 anemia

13 others

A8. **Do you smoking**？ A. Yes （smoking everyday and more than one cigarette per day） B. No

A8.1 If answered no, if you are a never-smoker? A. Yes B. No

A9. **Do you drinking？** A. Yes(drinking at least one time per weeke） B. No

A10. Do **physical activities_**________hours/week.

A11. **Sedentary time** __________ hours/day.

A12. **Watching TV _**_________ hours/day.

A13. **Number of drugment**

**Ability level scale**

|  | | | | | |
| --- | --- | --- | --- | --- | --- |
| **Mental Statu** | | | | | |
| **B.2.1 Cognition** | | | Test | | I say three things, repeat them, and remember, I'll ask you later "apple, watch, flag |
|  |  |  |  |  | Please draw a circular clock here and mark the clock at 10:45 |
|  |  |  |  |  | (2)Recall：“Now tell me, what are the three things I asked you to remember？”  Answer：_______、________、________（doesn't have to be in order） |
|  |  |  | Score  □ | | 0points，Draw the clock right（Draw a closed circle with the pointer in the correct position），And can recall 2-3 words |
|  |  |  |  |  | 1points，Draw the clock falsely or recall 0-1 word only |
|  |  |  |  |  | 2points，Cognitive impairment has been identified, such as alzheimer's disease |
| **B.2.2**  **Aggressive behaviour** | | | □ | | 0points，No physical aggression or verbal aggression |
|  |  |  |  |  | 1points，There are several physical attacks per month, or several verbal attacks per week |
|  |  |  |  |  | 2points，There are several physical attacks per week or verbal attacks per day |
| **B.2.3**  **Depressive symptom** | | | □ | | 0points，None |
|  |  |  |  |  | 1points，Depressed, silent, unwashed and inactive |
|  |  |  |  |  | 2points，Suicidal thoughts or behaviors existed |
| **B.2.4**  **Total Score** | | | □ | |  |
| **Sensory and communication** | | | | | |
| **B.3.1**  **Level of consciousness** | | | □ | | 0points，Be conscious and alert to your surroundings |
|  |  |  |  |  | 1points，Hypersomnia, characterized by excessive sleep duration. You can wake up when you call or push a patient's body，And can carry on the correct conversation or the execution instruction, stops the stimulation then continues to fall asleep |
|  |  |  |  |  | 2points，Lethargy, general external stimulation does not enable it to awaken，When given a strong stimulus, you can have a short period of conscious awareness. When you wake up, you can answer questions briefly |
|  |  |  |  |  | 3points，Coma, in a shallow coma when there is pain stimulus and pain expression; In a deep coma there is no response to the stimulus（If coma is assessed, severe disability is directly assessed and the following items may not be assessed） |
| **B.3.2** **Vision(wearing glasses)** | | □ | | 0points，Can read the standard font on the newspaper | |
|  |  |  |  | 1points，You can see a large font clearly, but you can't see the standard font on a newspaper | |
|  |  |  |  | 2points，Limited vision, unable to read newspaper headlines, but able to recognize objects | |
|  |  |  |  | 3points，It is difficult to identify objects, but the eye can follow them, seeing only light, color and shape | |
|  |  |  |  | 4points，Without vision, the eye cannot follow an object | |
| **B.3.3 Hearing** | | □ | | 0points，Can talk normally, can hear the sound of TV, telephone, doorbell | |
|  |  |  |  | 1points，Cannot hear clearly when speaking softly or more than 2 meters away | |
|  |  |  |  | 2points，It is difficult to communicate normally. You need to be quiet or speak loudly in a quiet circle before you can hear | |
|  |  |  |  | 3points，Only when the speaker speaks loudly or slowly can he hear part of it | |
|  |  |  |  | 4points，Out of hearing | |
| **B.3.4**  **Communication** | | □ | | 0points，Able to communicate with others without any difficulties | |
|  |  |  |  | 1points，Be able to express your needs and understand what others are saying，but need more time or help | |
|  |  |  |  | 2points，Difficulty in expressing needs or understanding | |
|  |  |  |  | 3points，Inability to express needs or understand what others are saying | |
| **B.3** **Sensory perception and communication ratings** | | □level | | 0：conscious，Vision and hearing were rated 0 or 1，communication score 0  1: conscious，But vision or hearing was rated 2, or 1 for communication.  2：conscious，But vision or hearing was rated 3, or 2 for communication.  Or sleepy, eyesight or hearing rating 3 and below, communication rating 2 and below  3: Conscious or sleepy，But at least one of them is 4 for vision or hearing, or 3 for communication. Or sleeping | |
|  | | | | | |
| **Social participant** | | | | | |
| **B.4.1**  **viability** | □ | 0points，In addition to personal care (such as eating, washing, dressing, and taking care of yourself), I can take care of household affairs (such as cooking, washing clothes) or manage my own affairs | | | |
|  |  | 1points，Apart from self-care in personal life, you can do housework, but you are not good at it, and family affairs are not well organized | | | |
|  |  | 2points，Self-care in personal life; You can only do some housework with the help of others, but the quality is not good | | | |
|  |  | 3points，Can take care of my own basic life affairs (such as diet and toilet). Can wash and gargle under supervision | | | |
|  |  | 4points，Personal basic life affairs (such as diet and convenience) require partial or total dependence on others | | | |
| **B.4.2**  **Operational capability** | □ | 0points，Skilled mental or physical skills can be carried out as usual | | | |
|  |  | 1points，The original proficiency of mental work or physical skills of the work ability has declined | | | |
|  |  | 2points，The original skilled mental work or physical skilled work is obviously not as good as before, part of the forgotten | | | |
|  |  | 3points，There are only a few fragments of skilled work to be retained and skills have been forgotten | | | |
|  |  | 4points，Obliteration of all previous knowledge or skills | | | |
| **B.4.3**  **Time/space orientation** | □ | 0points， Clear concept of time (day/year); Can go far alone, can grasp the orientation of the new environment quickly | | | |
|  |  | 1points，The concept of time is declining, the year, month, day clear, but sometimes several days away; You may go up and down the street alone, knowing the name and location of your present residence, but not knowing the way home | | | |
|  |  | 2points，The concept of time is poor, the year, month, day is not clear, we know the first half or the second half of the year; Can only move around the home alone, only known for the current residence, do not know the location | | | |
|  |  | 3points，he idea of time is very poor. The year, month and day are not clear. Can only be in the right and left neighbors round the door, do not know the name and location of the current residence | | | |
|  |  | 4points，No concept of time; You can't go out alone | | | |
| **B.4.4**  **Personal orientation** | □ | 0points，Know the relationship of people around you, know the meaning of terms like uncles, grandparents, aunts, nephews and nieces. Can distinguish the general age and identity of strangers | | | |
|  |  | 1points，Can identify the family relationship only, can't tell the general age of a stranger | | | |
|  |  | 2points，Can only call the family, or can only call follow others, do not know its relations, do not distinguish between generations | | | |
|  |  | 3points，Only know their family members who often live with them, can call children or grandchildren, can distinguished acquaintances and strangers | | | |
|  |  | 4points，Know only the protectors, can’t distinguished acquaintances and strangers | | | |
| **B.4.5**  **Social communication ability** | □ | 0points，Participate in the society, have certain adaptability in the social environment, treat people appropriately | | | |
|  |  | 1points，Being able to adapt to a simple environment and actively contact with people, it is difficult to find out the intelligence problems when meeting people at the first time and cannot understand metaphor | | | |
|  |  | 2points，Out of society, can be passive contact, will not be active to people, many uncomfortable words in conversation, easy to be cheated | | | |
|  |  | 3points，Can barely communicate with others, the speech was vague，with impertinent expression | | | |
|  |  | 4分，Difficult to make contact with people | | | |

**Interview guides**

1. Understand the content and purpose of the survey, and replace the content of the questionnaire with words that the elderly can understand.
2. About sensitive questions such as marital status, depression, suicidal tendencies, aggressive behavior, etc, we should seek help from social works or family members instead of directly ask participants.
3. Ability level standard

| Level | Criterion |
| --- | --- |
| Undamaged | ADL, mental states, sensory perception and communication are all rated 0, and social participation is rated 0 or 1 |
| Mild damaged | The ADL rated 0, but at least one of the mental state, sensory and communication level is 1 and above, or the social participation level is 2;  Or the ADL level is 1, at least one of the ratings of the mental state, sensory and communication and social participation is 0 or 1 |
| Moderate damaged | ADL was rated as 1, but mental states, sensory and communication, and social participation were all rated as 2, or one of those was rated as 3;  Or the ADL level is 2, and one or two of other domains was rated 1 or 2. |
| Severe damaged | The ADL was rated 3；  Or ADL, mental states, sensory and communication, social participation all were rated 2；  Or ADL was rated 2，and at least one category of mental state, sensory and communication, and social participation was rated 3. |
